# Trends in Antimicrobial Resistance at a Greek Tertiary Hospital over a 7-Year Period, Including the COVID-19 Pandemic

**DOI:** 10.3390/antibiotics14111067

**Published:** 2025-10-24

**Authors:** Eleni Mylona, Sofia Kostourou, Dimitroula Giankoula, Efthimia Spyrakou, Nektaria Michopanou, Chrysoula Kolokotroni, Maria Papagianni, Dimitris Kounatidis, Efstathia Perivolioti, Vasileios Papastamopoulos

**Affiliations:** 1 Fifth Department of Internal Medicine and Infectious Diseases, Evaggelismos General Hospital, 10676 Athens, Greece; iatreiomel@evaggelismos-hosp.gr (M.P.); vpapastamopoulos@evaggelismos-hosp.gr (V.P.); 2 Infection Control Committee, Evaggelismos General Hospital, 10676 Athens, Greece; kostour@med.uoa.gr (S.K.); nektariamix@nurs.uoa.gr (N.M.); enl@evaggelismos-hosp.gr (C.K.); 3 Center for Clinical Epidemiology and Outcomes Research (CLEO), 15451 Athens, Greece; dgiank@med.uoa.gr (D.G.); espyrakou@nurs.uoa.gr (E.S.); 4 Diabetes Center, First Propaedeutic Department of Internal Medicine, Medical School, National and Kapodistrian University of Athens, Laiko General Hospital, 11527 Athens, Greece; dimitriskounatidis82@outlook.com; 5 Microbiology Department, Evaggelismos General Hospital, 10676 Athens, Greece; perivolioti@yahoo.gr

**Keywords:** antimicrobial resistance, COVID-19 pandemic, MDR, XDR, PDR, ICU, Greece

## Abstract

**Background/Objectives**: Antimicrobial resistance (AMR) remains a major global threat, with the COVID-19 pandemic influencing its dynamics, although its overall impact remains uncertain. This study analyzed seven-year AMR trends, including the pandemic period, in a tertiary care hospital in Greece that served as a COVID-19 referral center. **Methods**: Multiresistant bacteria isolated from all biological specimens of hospitalized patients between January 2018 and December 2024 were recorded and classified as multidrug- (MDR), extensively drug- (XDR), or pandrug-resistant (PDR). Overall AMR was defined as the sum of these categories. Annual incidences of overall AMR, its categories, and predominant Gram-negative (*A. baumannii*, *K. pneumoniae*, *P. aeruginosa*) and Gram-positive [methicillin-resistant *S. aureus* (MRSA), vancomycin-resistant *Enterococcus* (VRE)] pathogens were analyzed for the entire hospital and by sector (medical, intensive care unit [ICU], surgical). Bloodstream infection (BSI) AMR was also evaluated. Trend analysis was performed using Joinpoint regression. **Results**: Overall AMR exhibited a transient peak around 2021 across the hospital, except in the surgical sector. A significant rise in average annual percentage change (AAPC) occurred only in the medical sector (*p* < 0.001). PDR incidence increased hospital-wide (*p* < 0.001). *K. pneumoniae*, *P. aeruginosa*, MRSA, and VRE rose significantly in the medical sector, whereas ICU incidences remained largely stable despite the 2021 peak. *A. baumannii* showed no significant change. BSI-related AMR increased in the medical sector (*p* < 0.001) but not in the ICU (*p* = 0.2). **Conclusions**: Although overall AMR did not rise uniformly, PDR organisms increased hospital-wide. These findings support updating empiric therapy guidelines, reinforcing infection prevention measures, and translating surveillance data into targeted stewardship actions to enhance patient care.

## 1. Introduction

Antimicrobial resistance (AMR) is a critical global health threat recognized by the World Health Organization (WHO). Gram-negative bacteria such as *Acinetobacter baumannii*, *Klebsiella pneumoniae*, and *Pseudomonas aeruginosa*—noted for their resistance to carbapenems—have been designated by the WHO as a top priority [1]. Their importance lies in their ability to transfer resistance genes and their association with severe, often fatal infection, with limited therapeutic options [1,2]. Among Gram-positive bacteria, methicillin-resistant *Staphylococcus aureus* (MRSA) and vancomycin-resistant *Enterococcus* (VRE) also remain prominent on the WHO bacterial priority pathogens list, as reaffirmed in its 2024 revision [1]. As emphasized in the WHO AMR action plans since 2015 [3], surveillance represents a cornerstone in controlling AMR [4]. Several surveillance programs worldwide have demonstrated that systematic monitoring of AMR can directly drive infection control and stewardship improvements. In Europe, data from the Italian AR-ISS surveillance system detected regional clusters of KPC-producing *K. pneumoniae*, leading to the introduction of national infection prevention guidelines and cohorting practices that cut carbapenem-resistant Enterobacterales (CRE) incidence by more than half within two years [5]. At the University Hospital of Crete, continuous AMR surveillance revealed escalating multidrug-resistant *A. baumannii* and *K. pneumoniae* infections. The subsequent restriction of carbapenem use and real-time prescriber feedback led to a 37% reduction in carbapenem consumption and a parallel decline in MDR infections [6]. Beyond Europe, the WHO GLASS pilot programs in Thailand and Kenya demonstrated that standardized AMR reporting and feedback to clinicians significantly reduced inappropriate antibiotic prescribing [7,8]. In the United States, NHSN surveillance triggered the CDC’s CRE Containment Response, achieving a 33% reduction in CRE transmission in targeted ICUs [9].

Surveillance becomes particularly critical when health systems are challenged by a pandemic. During the COVID-19 pandemic, several studies reported an increase in AMR infections [10,11,12]. A study across 243 U.S. hospitals found that hospital-onset AMR infections rose from 28.9 to 38.0 per 10,000 hospitalizations during the peak of the pandemic, representing a 31.5% increase. Although overall AMR rates returned to pre-pandemic levels as the pandemic waned, hospital-onset AMR remained elevated, indicating persistent challenges in infection control [10]. Similarly, a global review highlighted that the pandemic accelerated the emergence and transmission of AMR, particularly among Gram-negative organisms in hospital settings [11]. In the U.S., the Centers for Disease Control and Prevention (CDC) reported a 70% increase in infections caused by “nightmare bacteria” between 2019 and 2023, primarily due to the rise in carbapenem-resistant Enterobacterales (CRE) [13]. However, there are studies that do not confirm an association between the pandemic and AMR [14].

One of the factors contributing to this increase in AMR is the inappropriate or excessive use of antimicrobials, as well as the increased demand for ICU admission. Langford et al., in a meta-analysis, found that the likelihood of bacterial co-infection in patients presenting to the hospital with COVID-19 was low; nevertheless, the vast majority of patients received antimicrobials [15]. A study in Slovenia also observed changes in antibiotic consumption and the occurrence of AMR infections and colonization during the pandemic, underscoring the complex interplay between antimicrobial use and the development of resistance [16]. In the United States, a CDC report indicated that the pandemic disrupted progress in combating AMR, with over 29,400 deaths from AMR infections in 2020 alone. Despite increased awareness, the strain on healthcare systems during the pandemic led to difficulties in maintaining effective infection prevention and control measures [17].

The assessment of the exact magnitude of AMR remains challenging. According to the Antimicrobial Resistance Surveillance Report for Europe, published in 2023 and referring to isolates obtained in 2021, it appears that carbapenem resistance rates are high among *A. baumannii*, *K. pneumoniae*, and *P. aeruginosa*, with substantial regional variation [18]. A north-to-south and west-to-east gradient of resistance is evident for Gram-negative bacteria, with higher rates observed in the southern and eastern parts of the European region compared to the northern and western parts. Greece has long reported some of the highest rates of antimicrobial resistance (AMR) in the European Union, reflecting a complex interplay of epidemiological, healthcare, and policy-related factors. Carbapenem resistance exceeds 95% in *A. baumannii* and 50% in *K. pneumoniae* and ranges between 25 and 50% in *P. aeruginosa* [18,19]. Although the AMR gradient is less pronounced for Gram-positive bacteria, Greece nevertheless reports high resistance rates (25–50%) for both *MRSA* and *VRE* [18]. According to the European Centre for Disease Prevention and Control (ECDC, 2024) [20], multidrug-resistant organisms (MDROs) are widely endemic in Greek hospitals, with documented transmission even among newly admitted patients. Contributing factors include high antibiotic consumption in both community and hospital settings, suboptimal implementation of antimicrobial stewardship programs, and limited infection prevention and control (IPC) capacity due to resource constraints and high patient burden. Although legislative measures have reduced non-prescription antibiotic use, stewardship activities remain inconsistently applied across healthcare facilities. Furthermore, variations in laboratory diagnostic capacity and fragmented national coordination hinder comprehensive surveillance and timely response to emerging resistance threats [20].

The aim of the present study was to describe and analyze AMR trends over a seven-year period (January 2018–December 2024), encompassing the COVID-19 pandemic, in the largest tertiary care hospital in Greece, which served as a referral center for a substantial proportion of COVID-19 patients, in an attempt to shed light on whether the pandemic ultimately affected the dynamics of AMR.

## 2. Results

### 2.1. Description of the Clinical Isolates

**Figure 1:**
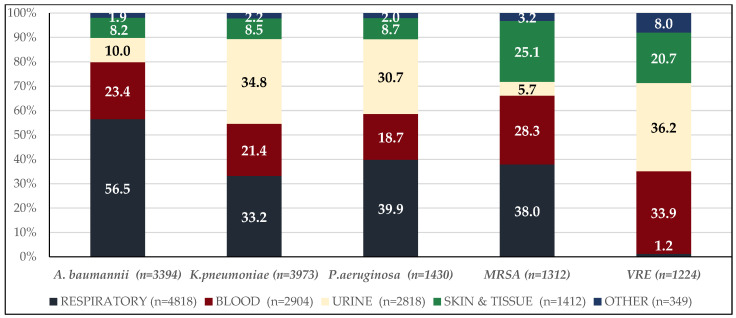
Distribution of organisms (n = 12,321) by culture site.

Over the seven-year period, 12,321 multiresistant bacterial isolates were recovered from 456,469 hospitalized patients, corresponding to 2,051,688 patient-days. Respiratory specimens accounted for 4812 (39.2%) isolates, followed by blood, at 2904 (23.6%), urine, at 2818 (22.9%), and skin/tissue samples, at 1412 (11.5%), with other sources, including cerebrospinal, pleural, and peritoneal fluids, comprising 349 (2.8%) (Figure 1). Gram-negative isolates, of which there were 9764 (79.35%), predominated over Gram-positive ones, of which there were 2537 (20.64%). The annual distribution of resistant pathogens is presented in Table 1. Among all resistant isolates, 4788 (38.9%) were MDR, 5515 (44.8%) XDR, and 1998 (16.2%) PDR. Regarding XDR Gram-negative isolates, 1394 (25.3%) accounted for *A. baumannii*, 2941 (53.3%) for *K. pneumoniae*, 1154 (20.9%) for *P. aeruginosa*, and the remaining 26 isolates (0.5%) for other Enterobacterales. Among PDR Gram-negative isolates, 1724 (86.3%) accounted for *A. baumannii*, 187 (9.4%) for *K. pneumoniae*, and the remaining 87 isolates (4.4%) for *Providencia stuartii*.

### 2.2. Trends in Total AMR in the Entire Hospital and per Sector

**Figure 2:**
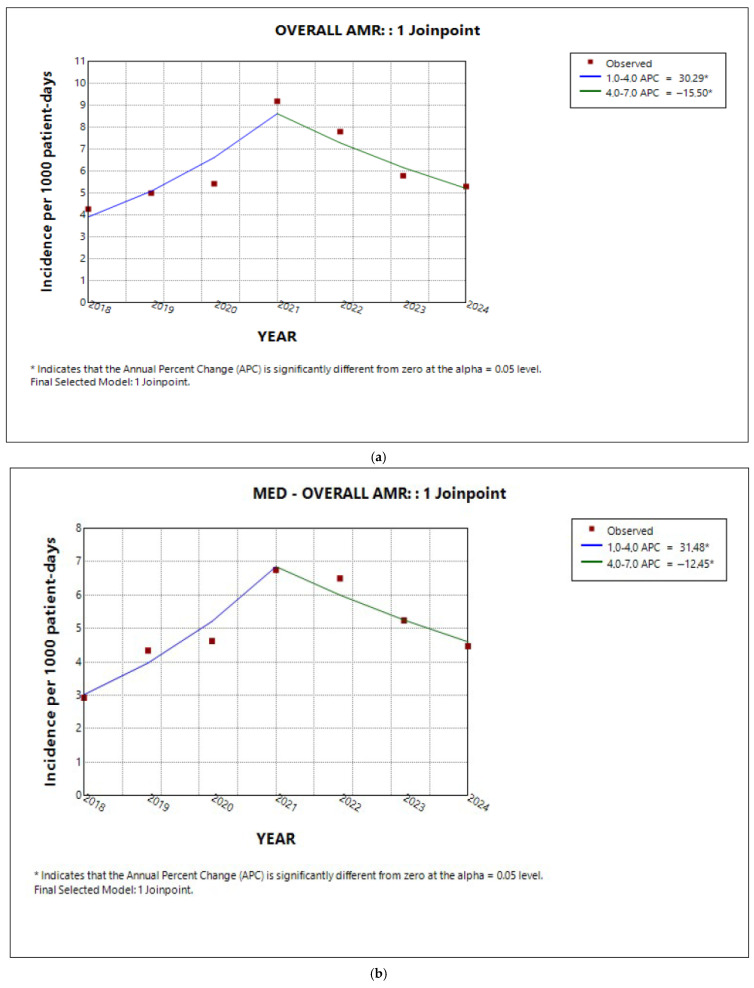

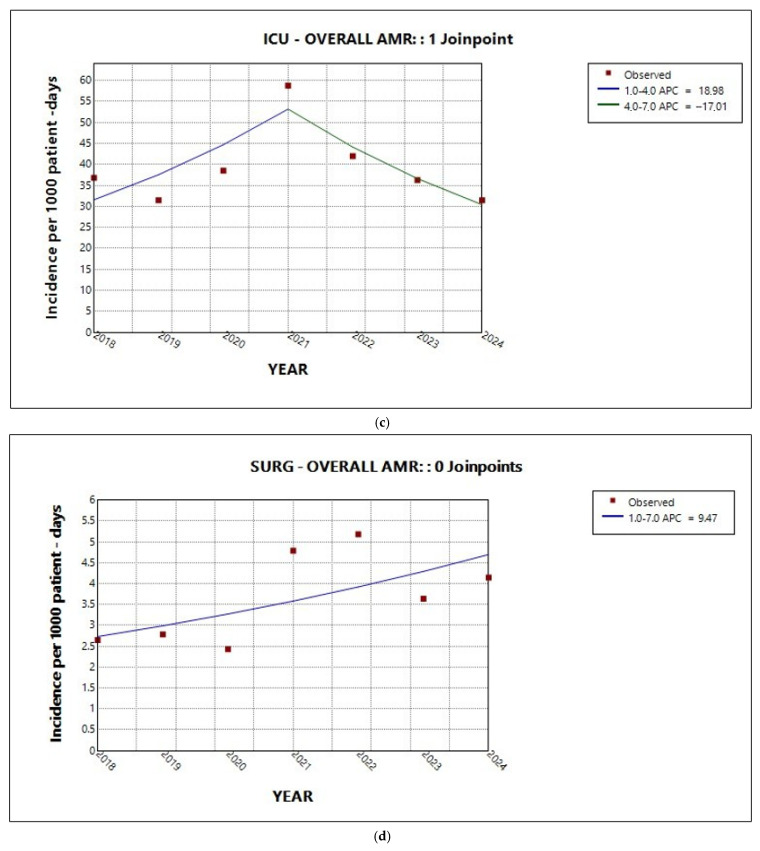
Trend in overall AMR in (**a**) the entire hospital, (**b**) the medical sector, (**c**) the ICU, and (**d**) the surgical sector during the studied period.

Across the entire hospital, the annual trend in overall AMR followed the pattern shown in Figure 2a. According to this data, the incidence of AMR increased annually from 2018 to 2021 (APC 30.6%, 95% CI 15.7 to 64.5%, *p* < 0.001) and then decreased annually till 2024 (APC 15.4%, 95% CI −30.8 to −5.5%, *p* < 0.001). Although the annual changes observed during 2018–2021 and 2021–2024 were statistically significant, the average annual percentage change (AAPC) for the overall study period did not reach statistical significance (AAPC 4.9%, 95% CI −1.3 to 11.9%, *p* = 0.1).

When the analysis was performed separately for the three hospital sectors (medical, ICU, surgical), it was evident that the overall AMR followed the same trend pattern, with a transient increase around 2021 in both the medical sector (Figure 2b) and the ICU (Figure 2c), while in the surgical sector (Figure 2d), the AMR trend slightly increased, although still to a statistically non-significant degree (APC 9.8%, 95% CI −1.8 to 23.3%, *p* = 0.1).

Although an increase–decrease pattern was observed in the APCs for the three sectors, the incidence trend of AMR according to the AAPC over the 7-year study period in the medical sector only demonstrated a statistically significant upward trend. Time trends indicated by average annual percent changes (AAPCs), 95% confidence intervals (CIs), and *p* values are presented in Table 2.

### 2.3. Trends in Incidence by Resistance Category

**Figure 3:**
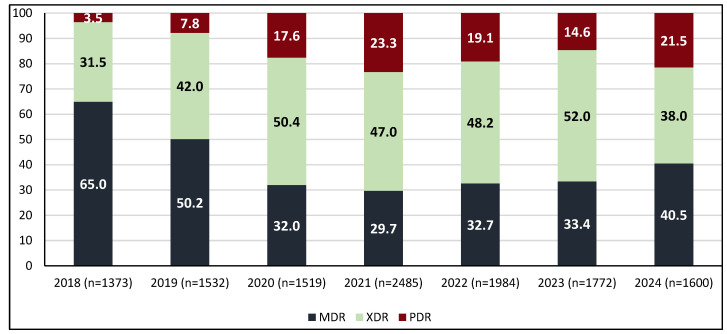
The annual percentage distribution of resistance phenotypes.

**Figure 4:**
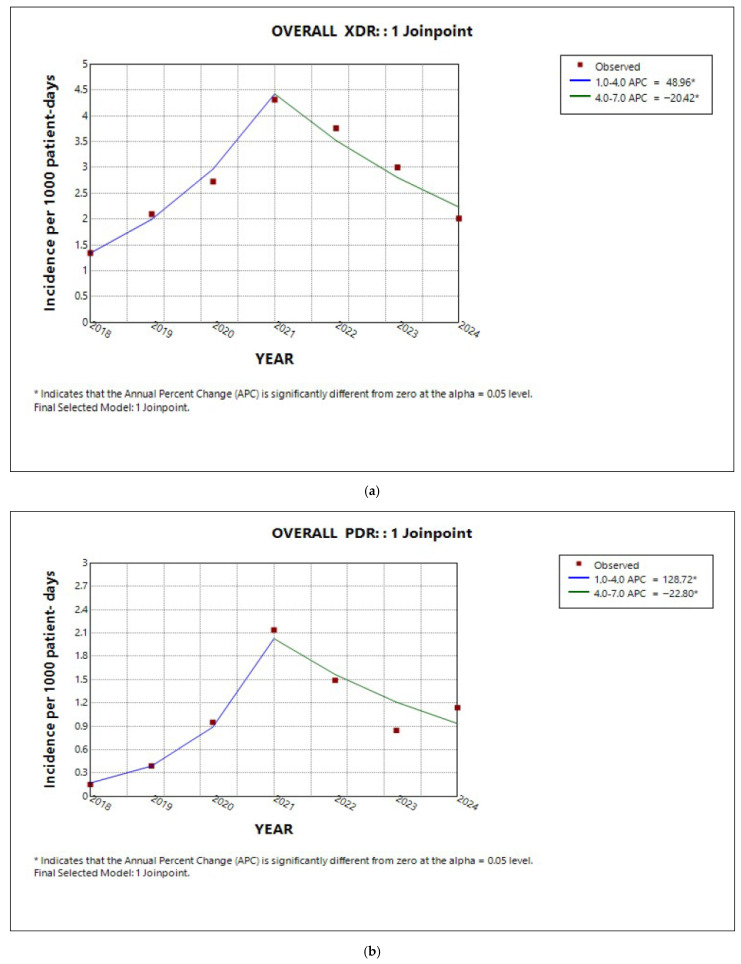

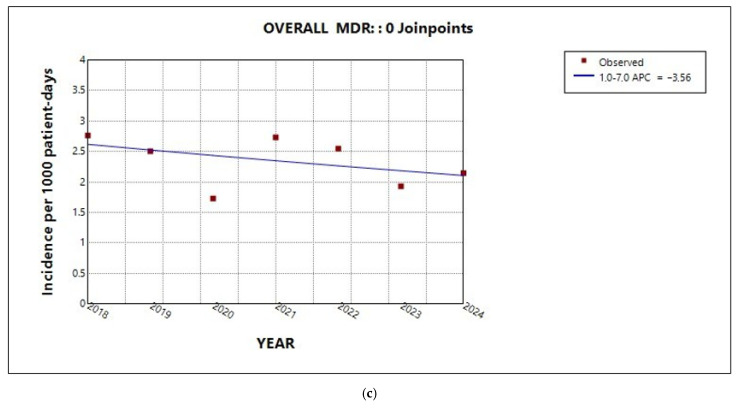
Trends in incidence of (**a**) XDR, (**b**) PDR, and (**c**) MDR in the entire hospital during the studied period.

The annual percentage distribution of resistance categories is shown in Figure 3, where a yearly decrease in MDR is observed, accompanied by a corresponding increase in XDR and PDR throughout the 7-year study period. When the trends were evaluated statistically, the observations were as follows:

For the entire hospital, the annual trends of XDR and PDR mirrored that of total AMR, as described in the previous paragraph. During 2018–2021, both XDR and PDR increased (APC 48.9%, 95% CI 25.3 to 97.4%, *p* < 0.001; APC 128.7% 95% CI 88.9% to 215.8%, *p* < 0.001, respectively), followed by a subsequent decline (APC −20.4%, 95% CI −35.4% to −6.9% *p* = 0.004; APC −22.8%, 95% CI −34.8% to −11.3%, *p* = 0.0008, respectively) (Figure 4a,b). Notably, despite this ‘increase–decrease’ pattern, the incidences of XDR, and even more markedly of PDR, were estimated to have increased at a statistically significant level over the entire 7-year study period [(AAPC 8.9%, 95% CI 0.16 to 20.5%, *p* = 0.04) and (AAPC 32.8%, 95% CI 21.8 to 53.08%, *p* < 0.001), respectively]. MDR exhibited a slight declining trend over the 7-year study period (Figure 4c); however, the decrease was not statistically significant (AAPC −3,6%, 95% CI −11.8 to 4.4%, *p* > 0.05).

**Figure 5:**
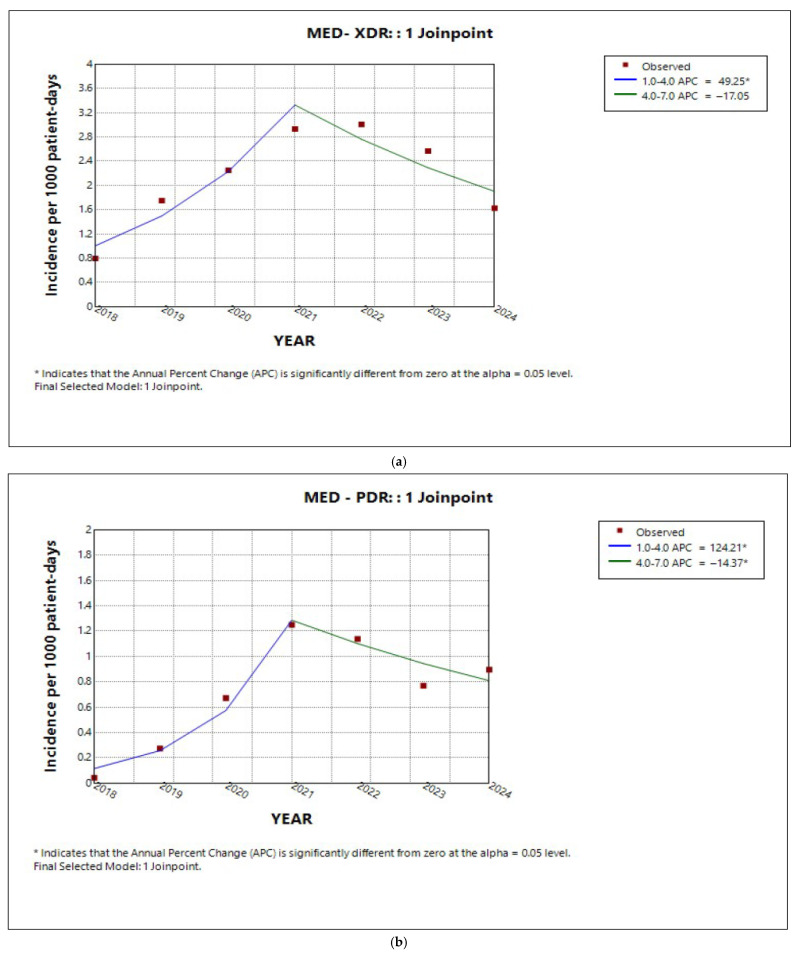

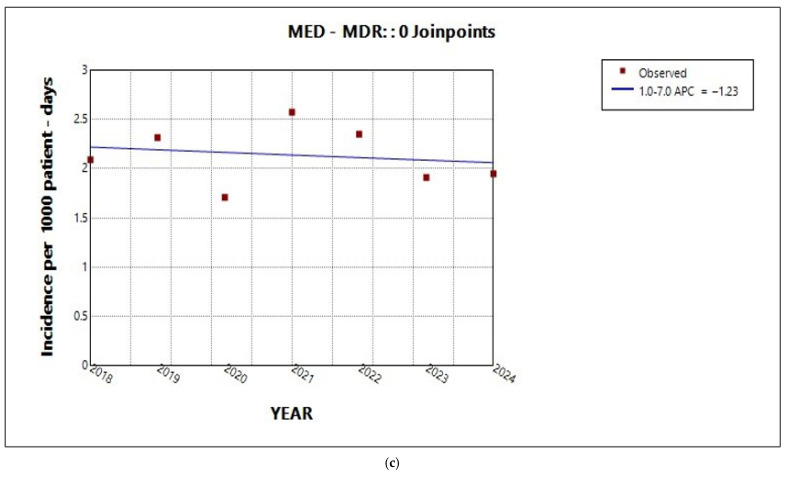
Trends in incidence of (**a**) XDR, (**b**) PDR, and (**c**) MDR in the medical sector during the period studied.

**Figure 6:**
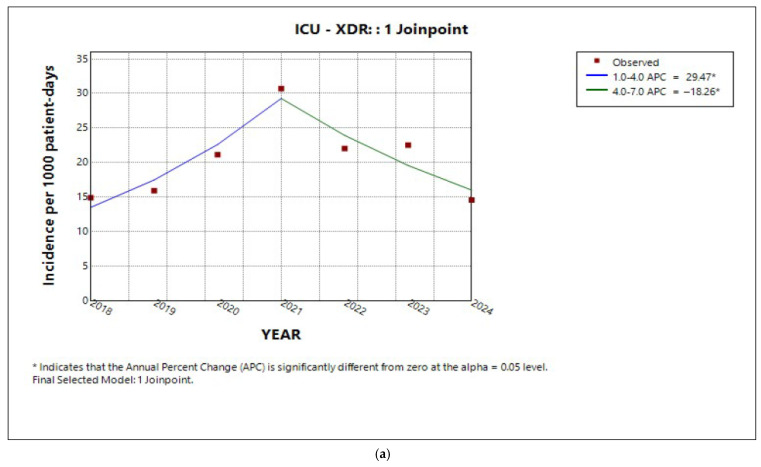

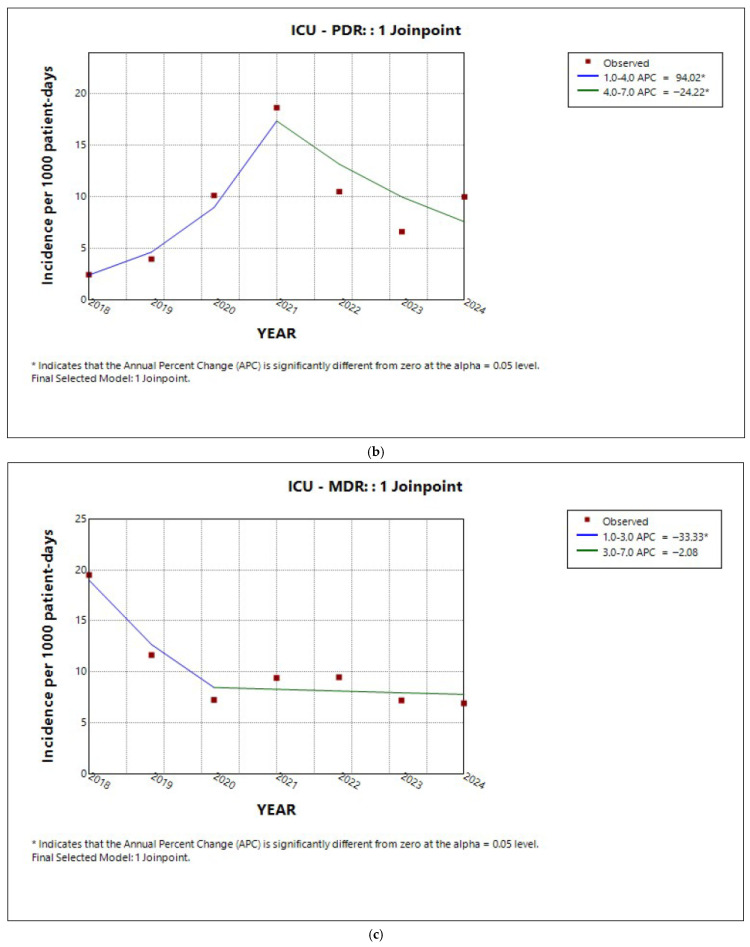
Trends in incidence of (**a**) XDR, (**b**) PDR, and (**c**) MDR in the ICU during the period studied.

In the medical sector, as well as in the ICU, the analysis revealed an ‘increase–decrease’ pattern in the annual percentage change in incidence for both XDR and PDR (Figure 5a,b and Figure 6a,b, respectively). However, the average annual percentage change over the entire study period was estimated to be significantly increasing only for PDR for both sectors (AAPC 38.6%, 95% CI 5.3 to 47.4%, *p* = 0.007; AAPC 21.2%, 95% CI −5.2 to 65.1%, *p* < 0.001, respectively). Similarly to MDR in the entire hospital, the incidence trend in the medical sector slightly decreased (Figure 5c), without reaching statistical significance (APC and AAPC −1.2%, 95% CI −11.7 to 10.8%, *p* = 0.7). In contrast, MDR incidence in the ICU declined by 33.3% annually until 2020 (APC 33%, 95% CI −45.5 to −16.1%, *p* < 0.001), after which it remained relatively stable (APC −2.08%, 95% CI −15.5 to 28.7%, *p* = 0.9) (Figure 6c). Overall, the average annual percentage change in MDR incidence in the ICU over the 7-year period was evaluated as significantly declining (AAPC −13.85%, 95% CI −20.5 to −7.6%, *p* < 0.001).

**Figure 7:**
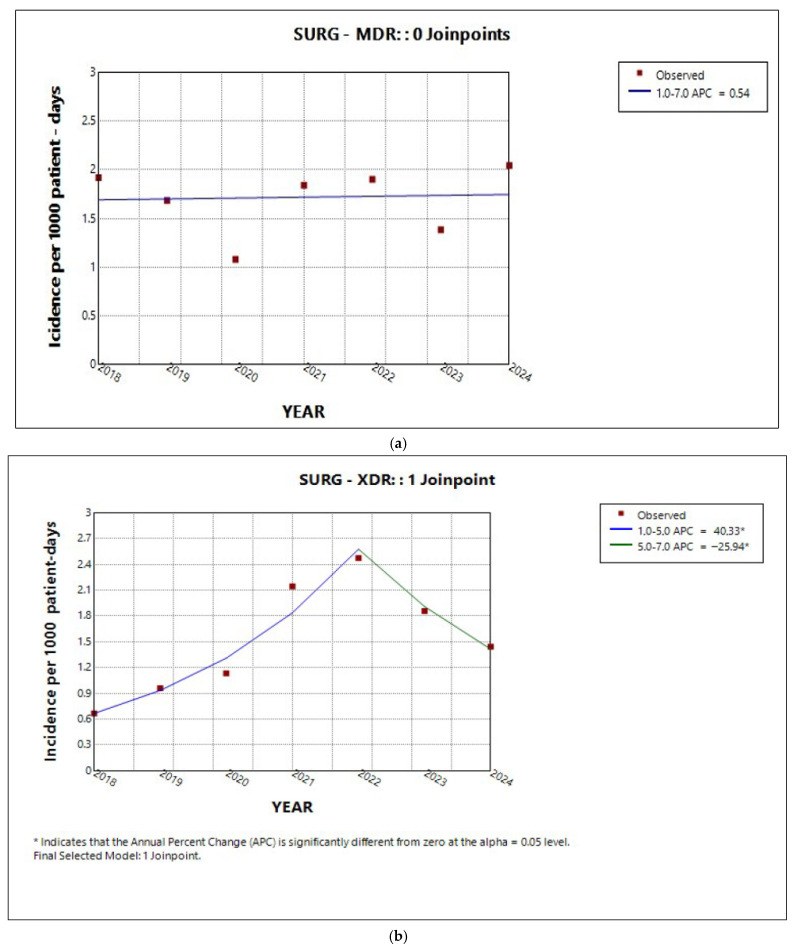

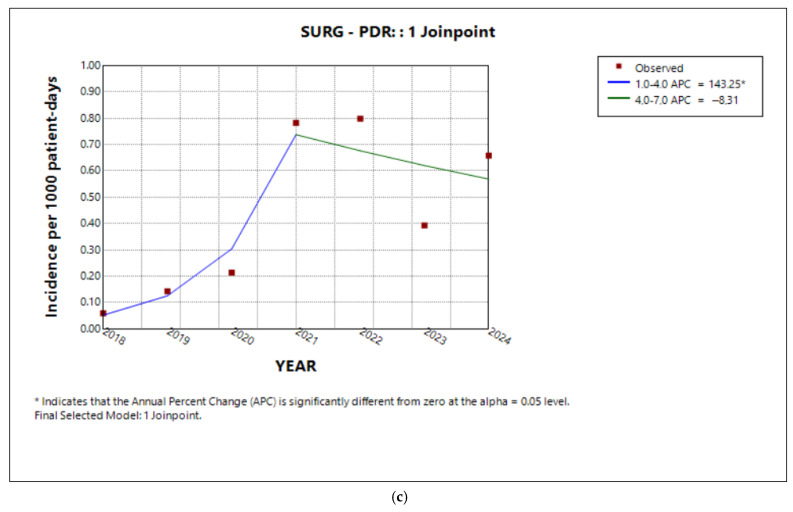
Trends in incidence of (**a**) MDR, (**b**) XDR, and (**c**) PDR in the surgical sector during the period studied.

In the surgical sector, while MDR rates remained stable (Figure 7a) throughout the 7-year study period (AAPC 0.53%, 95% CI −7.0 to 7.9%, *p* = 0.9), XDR and PDR rates followed the increase–decrease pattern (Figure 7b,c). Both depicted a significant average annual percentage increase for the entire study period (AAPC 13,4%, CI 9.15 to 18.9%, *p* < 0.001; AAPC 40.34%, 95% CI 31.2 to 85.9%, *p* < 0.001, respectively).

### 2.4. Incidence Trends by Bacterial Species

**Figure 8:**
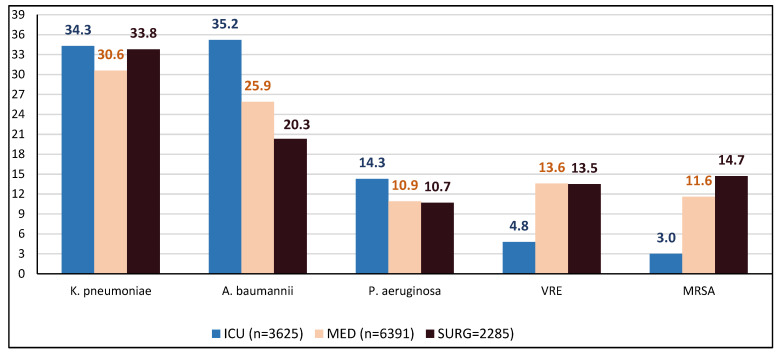
The percentage distribution of resistance of the major pathogens by sector.

The percentage distribution of the resistance of the major pathogens by sector is shown in Figure 8, where it can be observed that in the ICU, *K. pneumoniae* and *A. baumannii* were the predominant pathogens, followed by *P. aeruginosa*, whereas *MRSA* and *VRE* were rarely detected. In medical and surgical wards, *K. pneumoniae* predominated, followed by *A. baumannii*, while *MRSA* and *VRE* were more frequently isolated compared to the ICU.

**Figure 9:**
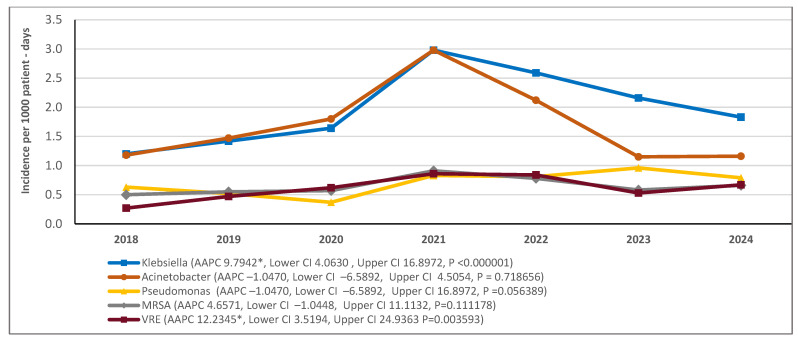
Trends in incidence of the most frequently isolated bacteria. Time trends indicated by average annual percent changes (AAPCs), 95% confidence intervals (CI), and *p* values. * indicates that AAPC is significantly different from zero at the alpha=0.05 level.

Analysis of the annual incidence trends of the bacteria in the entire hospital showed that *A. baumannii*, *K. pneumoniae*, and *MRSA* significantly increased and then declined around 2021, whereas *VRE* significantly increased, with a subsequent insignificant decrease. *P. aeruginosa* remained nearly stable (Table 3). However, when the average annual percentage change in incidence was assessed over the 7-year period, *K. pneumoniae* and *VRE* were the only pathogens that showed a statistically significant increase in the hospital (Figure 9).

**Figure 10:**
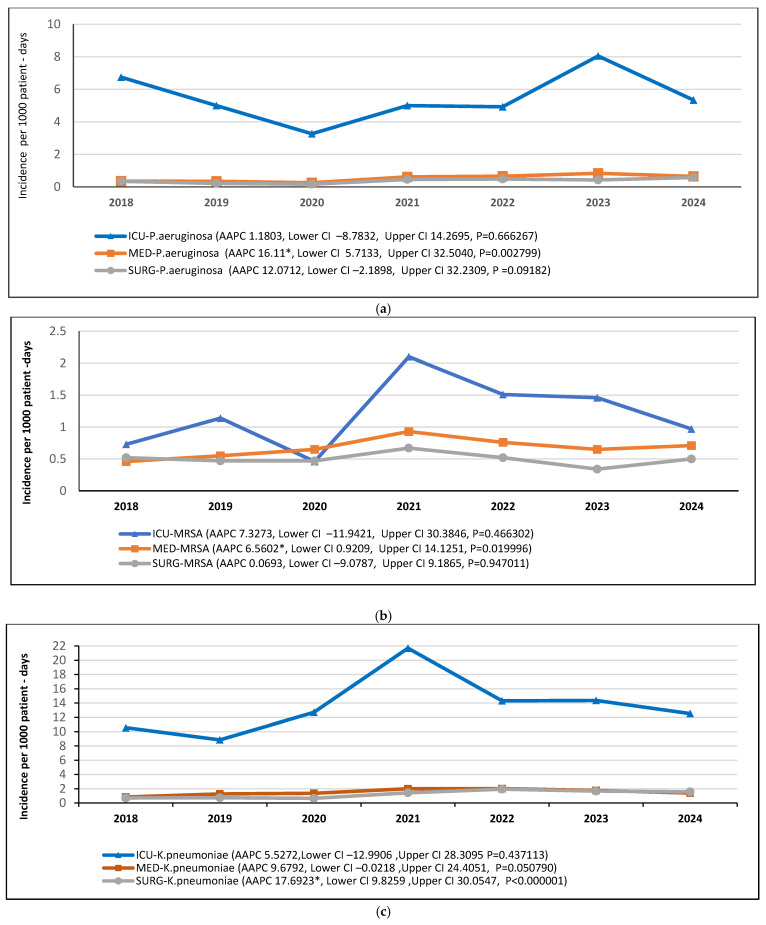

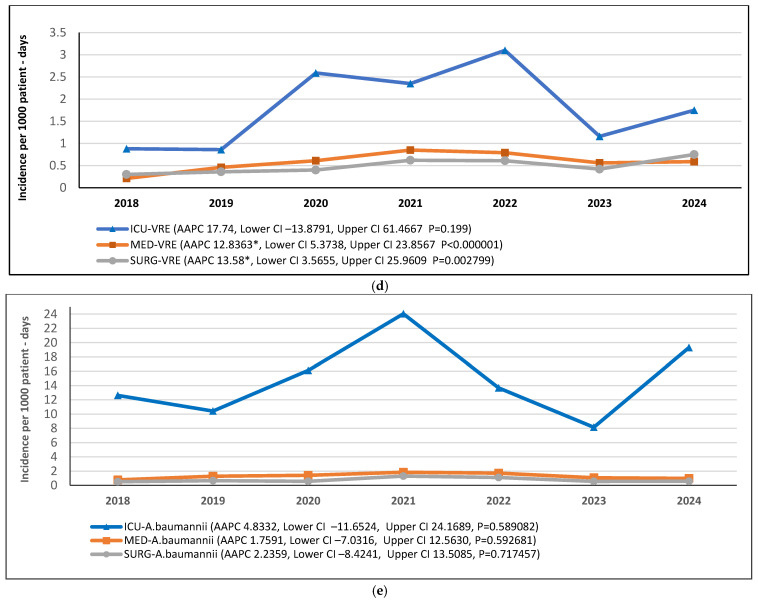
Trends in incidence of (**a**) *P. aeruginosa*, (**b**) *MRSA*, (**c**) *K. pneumoniae*, (**d**) *VRE*, and (**e**) *A. baumannii* in each hospital sector (ICU, medical, surgical). Time trends indicated by average annual percent changes (AAPCs), 95% confidence intervals (CI), and *p* values. * indicates that AAPC is significantly different from zero at the alpha = 0.05 level.

When the incidence trend of each bacterial species was examined by sector, it was found that, during the study period, the incidence of *P. aeruginosa* and *MRSA* were increased significantly only in the medical sector, whereas *K. pneumoniae* and *VRE* were increased significantly in both medical and surgical sectors (Figure 10a–d). *A. baumannii* did not change significantly in any sector (Figure 10e). Interestingly, in the ICU, the incidence of all pathogens did not change significantly throughout the study period, despite the transient increase observed around 2021 (Figure 10).

### 2.5. Trend in Total AMR in Isolates from Blood Cultures

**Figure 11:**
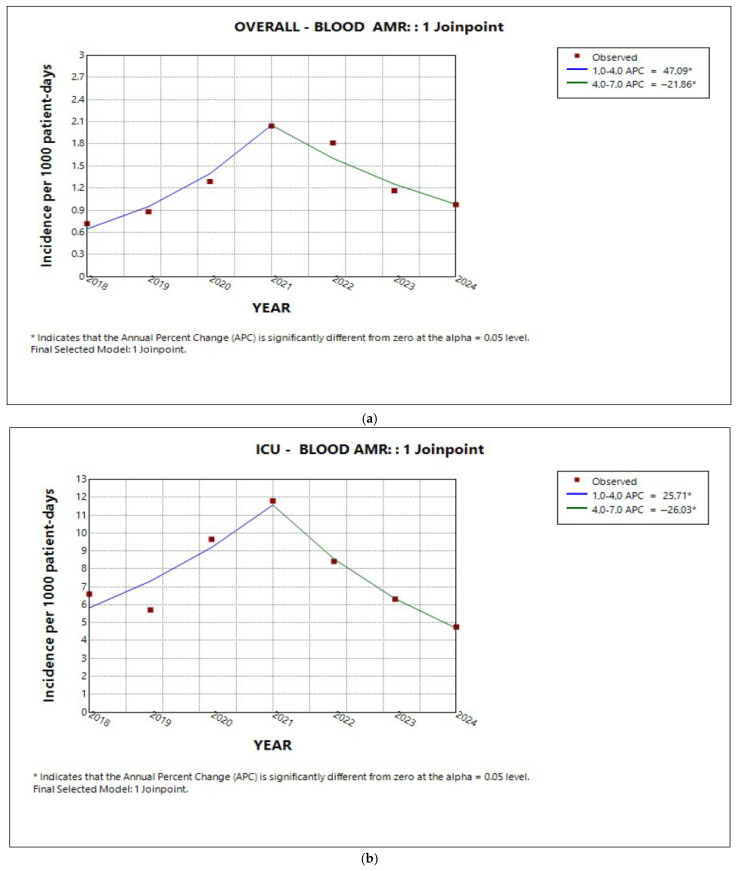

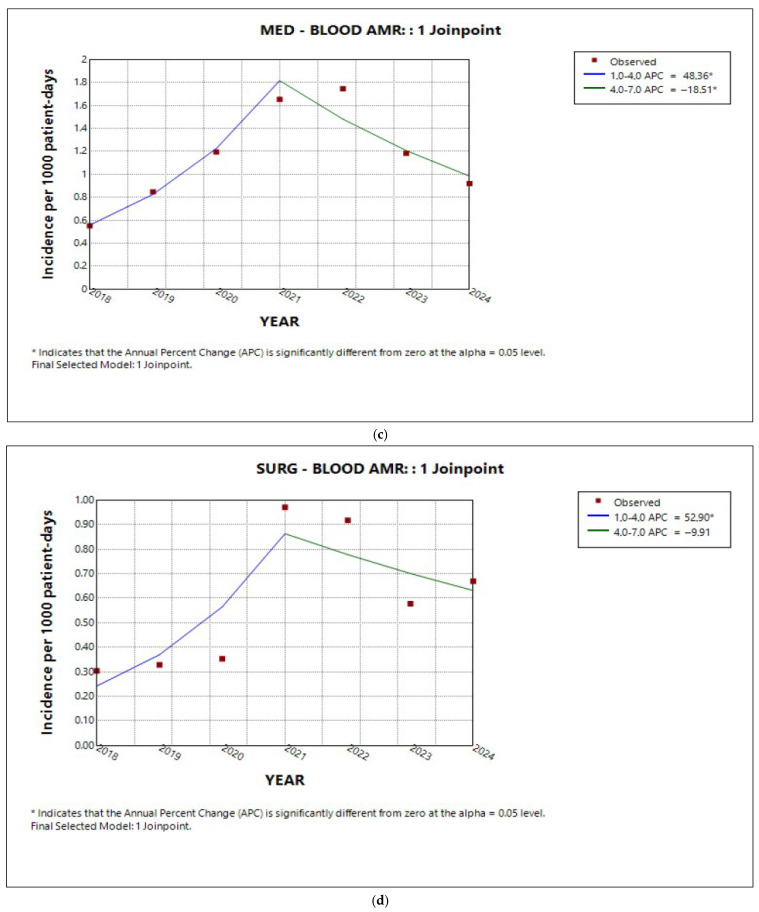
Trends in incidence of the bloodstream AMR (**a**) in the entire hospital, (**b**) in the ICU, (**c**) in the medical sector, and (**d**) in the surgical sector.

Since positive blood cultures are considered a reliable indicator of infection, we also examined the incidence trend of overall AMR in blood cultures for the entire hospital and by sector, where a similar ‘increase–decrease’ distribution pattern around 2021 was observed (Figure 11a). However, only the ICU, following the peak observed in 2021, appeared to return the incidence of bloodstream infection resistance to pre-COVID-19 levels, as reflected by the lack of a significant change in the average annual percentage of AMR incidence, in contrast to the medical and surgical sectors (Figure 11b–d).

Incidence trends of overall AMR in blood culture isolates over the 7-year study period were estimated for the entire hospital and by hospital sector. Time trends were indicated by average annual percent changes (AAPCs), 95% confidence intervals (CIs), and *p* values and are presented in Table 4.

## 3. Discussion

This study aimed to characterize trends in overall AMR, its categories (MDR, XDR, PDR), and in the most common bacterial isolates across the entire hospital and by sector (medical, ICU, surgical), as well as the sector-specific AMR trends in blood culture isolates over a seven-year period that encompasses the pandemic years. Our main findings are as follows: (1) Overall AMR showed an “increase–decrease” pattern around 2021 across the hospital and by sector, except in the surgical sector, without fully returning to pre-COVID-19 levels. In the medical sector, which included COVID-19 units, the increase was statistically significant over the study period. (2) While AMR rose significantly only in the medical sector, all sectors showed qualitative changes, with PDR significantly increasing. (3) Incidence of *K. pneumoniae*, *P. aeruginosa*, *MRSA*, and *VRE* increased significantly in the medical sector, whereas in the ICU, pathogen incidence remained largely unchanged despite the transient 2021 peak. *A. baumannii* remained stable in the entire hospital and by sector. (4) Bloodstream infection AMR mirrored this pattern, with the medical sector showing an increase in blood AMR, whereas the ICU did not.

The COVID-19 pandemic imposed conditions that theoretically influenced AMR [17]. During this period, measures were implemented that enhanced individual and environmental protection both in the community and in hospitals, promoted social distancing, and reduced travel and hospital admissions for non-COVID-19 causes and elective invasive procedures, factors that could potentially contribute to limiting AMR [21,22]. At the same time, however, the pandemic led to prolonged hospitalizations, overprescription of antibiotics due to excessive concern about secondary bacterial infections, and increased admissions to intensive care units [23,24,25]. Notably, the special report released by the CDC in 2022 on the impact of COVID-19 on AMR highlighted violations of infection control procedures prompted by increased workload—such as hand hygiene, equipment cleaning, patient separation, and the use of personal protective equipment—hindering the fight against AMR [17].

Despite the publication of several studies on AMR trends during the COVID-19 era [26,27,28,29,30,31], subsequent meta-analyses have shown that the available data are inconsistent and sometimes contradictory, largely due to methodological and geographical heterogeneity [12,15,32]. Specifically, some studies focus on prevalence, while others examine incidence [12], and certain analyses include all isolates from any site, whereas others restrict their evaluation to blood culture isolates only [33]. Moreover, most available studies rely on data up to 2021, coinciding with the peak of the first wave of the pandemic [12,15,26,27,28,29,30,31,32], without providing an indication of the trajectory of AMR after the end of the pandemic. Despite this heterogeneity, evidence suggests that AMR increased during the COVID-19 period compared with the pre-COVID-19 era, with the effect being particularly pronounced in intensive care units [12,15,32], although some reports do not support this finding [11,27,34,35]. In this context, our finding of a significant year-on-year increase in AMR up to 2021, both hospital-wide and within the medical sector, is consistent with the literature and likely reflects the fact that, during the pandemic, our hospital predominantly admitted COVID-19 patients in dedicated COVID-19 units, which were included within the medical sector, as defined in the present study. Interestingly, AMR in the surgical sector, which was largely scaled down during the pandemic due to bed reductions and limitation of procedures to urgent cases only, did not appear to be affected at all.

One unique characteristic of the present study is the demonstration of the qualitative shift in AMR, with a significant increase in both XDR and PDR across the hospital overall and in PDR within each sector (medical, ICU, and surgical). Two points merit particular attention: first, although the incidence of XDR and PDR declined after their peak in 2021, they have not yet returned to the levels observed in 2018, resulting in their average annual percentage over the seven-year study period being evaluated as increasing. Second, even in the sectors where overall AMR did not change significantly (ICU and surgical) during the study period, the rise in PDR was considerable. In the literature, studies addressing the trends of antimicrobial resistance categories are relatively scarce [27,36,37,38]. Pascal et al., in a study evaluating the incidence of carbapenem-resistant *Enterobacteriaceae* (CRE) and carbapenem-resistant *A. baumannii* (CRAB) in isolates from all sites of ICU patients, reported no significant change in the incidence of CRE between 2019 and 2020. They did, however, observe a decrease in the prevalence of KPC in favor of OXA-48- and VIM-producing strains, providing insight into a qualitative shift in AMR during the pandemic even in the absence of quantitative changes [27]. In Egypt, Abdelmoneim et al. reported a significant increase in the prevalence of MDR and XDR strains of *A. baumannii* and *K. pneumoniae* during the pandemic compared with the pre-COVID-19 period [37].

In Greece, Lagadinou et al. analyzed the prevalence of XDR over an eight-year period (2016–2023) in blood culture isolates. According to their findings, the prevalence of XDR *A. baumannii* increased significantly from 2016 to 2019 and subsequently stabilized, indicating that XDR *A. baumannii* was already endemic in that hospital prior to the onset of COVID-19. They also reported a decline in the prevalence of XDR *K. pneumoniae* from 2019 to 2022, without providing data on MDR and PDR trends to know in favor of which resistance category the decrease was [36]. By contrast, Tsalidou et al., examining the prevalence trends of AMR and its categories in a 227-bed regional Greek hospital from 2018 to 2023, found a statistically significant increase in the prevalence of MDR and PDR strains and a non-significant rise in XDR. The discrepancy between their findings and ours can likely be attributed, first, to the use of CLSI breakpoints for resistance interpretation in their study, second, to the inclusion of ambulatory patients in the study, and, third, to the fact that their data were derived from a smaller hospital managing fewer and potentially less severe cases [38].

The fact that reports of a qualitative shift in AMR toward XDR and PDR in the post-pandemic era come from regions such as Egypt, Greece, and Italy support the observation of a north-to-south gradient of resistance in the WHO European Region [18]. Specifically for Greece, where MDR pathogens were already endemic before the pandemic, the epidemiological pressure resulting from increased antimicrobial use during the COVID-19 period, overcrowding in hospital wards, higher rates of ICU admissions, and reduced adherence to infection prevention and control (IPC) measures could have contributed to a qualitative shift in AMR. This shift has enormous consequences for daily clinical practice. The selection of empirical antimicrobial therapy becomes even more challenging, while limited or delayed access to certain newer beta-lactam antibiotics, which are often the only effective therapeutic options, leads to the use of combination regimens of questionable efficacy. This, in turn, adversely affects patient prognosis [39,40] and perpetuates the vicious cycle of selective pressure and resistance maintenance.

In the analysis of AMR trends by bacterial species, a key finding was that while all four organisms—*K. pneumoniae*, *P. aeruginosa*, *MRSA*, and *VRE*—showed a significant increase in the medical sector, no such rise was observed in the ICU throughout the study period, despite the transient peak around 2021. Similarly, bloodstream infection AMR increased in the medical sector, while no such rise was observed in the ICU. The increase observed in the medical sector may be partly explained by the inclusion of COVID-19 units. By contrast, in ICUs, which also admitted COVID-19 patients, mostly in more severe condition, such an increase was not observed. Several factors may account for the absence of a sustained increase. ICUs are smaller, closed units with a better nurse-to-patient ratio and their personnel are typically more experienced and better trained in infection prevention and control practices. Our hospital ICU, specifically, has previously participated in international projects focused on hand hygiene and the prevention of catheter-related bloodstream infections (the PROHIBIT project, 2011–2013) and, since 2021, in the Greek Infection Prevention Program (GRIPP), which aims to raise awareness among healthcare workers across Greece in infection prevention and control principles with the goal of reducing hospital-acquired infections and AMR. It is plausible that these principles were applied more rigorously, enabling ICUs to limit the spread of resistant organisms and to return to baseline levels following the temporary increase in 2021. Finally, the absence of any trend in *A. baumannii* incidence both at hospital level and across sectors is consistent with its established endemic presence, which may overshadow temporal fluctuations.

The main strengths of this study are its long observation period of seven years, including almost two years after the official end of the COVID-19 pandemic, which provides a clearer picture of its impact on AMR over time beyond the increases reported in preliminary studies. It also includes data from a variety of clinical specimens, not just blood cultures, enabling a broader assessment of AMR. Finally, it applies the ECDC classification of MDR, XDR, and PDR, enabling detection of qualitative shifts in resistance patterns. Limitations include the absence of clinical data to distinguish infection from colonization and community-acquired from hospital-acquired infection and its single-center design, although results from this large tertiary hospital generally reflect national surveillance trends (WHONET-Greece). Unfortunately, we do not have data regarding IPC practices in the hospital departments, antibiotic consumption, or ward overcrowding in order to compare them with the incidence of resistant pathogens and investigate a possible association between them. The absence of clinical data prevented us from assessing differences in patient outcomes according to whether they had bacteremia caused by MDR, XDR, or PDR pathogens, which is an additional limitation.

Despite the aforementioned limitations our study provides valuable results on the impact of COVID-19 on AMR. Our findings are consistent with the WHO’s 2024 prioritization, which identifies *A. baumannii*, *K. pneumoniae*, and *P. aeruginosa* resistant to carbapenems, as well as MRSA and VRE, as top priorities [1]. The example of the ICU, which managed to limit the surge of AMR during the pandemic through the strict application of IPC principles, clearly shows that these measures must also be implemented throughout the rest of our hospital. However, the hospital is not the only setting where the agent and host come into contact, completing the epidemiological triad that leads to infection spread [41]. Resistant microorganisms exist in humans, animals, food, and the environment. “One Health” is now a term used to describe the principle recognizing that human, animal, and environmental health are interconnected; therefore, AMR must be addressed not only in humans but also in animals, plants, food, and the environment [42,43]. This makes AMR a complex epidemiological issue that also does not respect borders. This means that no individual member of the European Union—or globally—can tackle the problem on its own, and no member can claim that the issue does not concern them. Therefore, surveillance studies such as this one are essential to demonstrate the magnitude of the problem and identify trends that can both support the updating of local empirical guidelines and guide European and global efforts to combat AMR.

## 4. Materials and Methods

This was a retrospective surveillance study conducted in Evaggelismos General Hospital, a 946-bed tertiary hospital in Greece. The study interval was 7 years, from January 2018 to December 2024. During this period, we investigated the incidence of resistant bacteria.

The investigation included bacteria isolated from any biological sample taken for diagnostic purposes from hospitalized patients, without having the clinical information to discriminate infection from colonization. Positive cultures drawn within 48 h from patients’ admission were also included. For each patient, only the first specimen was included. The site of collection was categorized as blood, respiratory, urine, skin and tissue, or other. When multiple samples were obtained per patient on the same day, the isolate recovered from a sterile site was selected. Then, we classified bacteria based on their resistance phenotype to multidrug-resistant (MDR) organisms when they were non-susceptible to at least one agent in three or more antibiotic categories, extensively drug-resistant (XDR) when non-susceptible to all but one or two antibiotic categories, and pandrug-resistant (PDR) when they had developed resistance to all agents in all antibiotic categories, according to the standardized terminology [44]. Overall AMR was considered as the sum of MDR, XDR, and PDR isolates. In order for our results to be comparable with those of other studies, the classification of resistant Gram-negative bacteria as MDR, XDR, and PDR did not consider the susceptibility testing of newer antibiotics (ceftolozane/tazobactam, ceftazidime/avibactam, imipenem/cilastatin/relebactam, and meropenem/vaborbactam), since their systematic reporting in the sensitivity test by the laboratory only started in the spring of 2023.

We first compared the annual incidence of AMR in the hospital, followed by analyses for each resistance category (MDR, XDR, and PDR), as well as for the predominant Gram-negative (*A. baumannii*, *Klebsiella pneumoniae*, and *P. aeruginosa*) and Gram-positive bacteria (*MRSA* and *VRE*). The same analyses were performed for resistant isolates recovered from blood cultures. Analyses were conducted both for the hospital as a whole and by sector (medical, surgical, and ICU). The medical sector comprised Internal Medicine, Cardiology, Nephrology and the Kidney Transplant Unit, Neurology, Hematology–Oncology, and the Hematopoietic Stem Cell Transplantation Unit. The COVID-19 ward, which operated from March 2020 to December 2022, was classified under the medical sector. The surgical sector included General Surgery, Orthopedics, Neurosurgery, Urology, Maxillofacial Surgery, Ear–Nose–Throat Surgery, Cardiothoracic Surgery, and Vascular Surgery. The hospital also had three adult intensive care units (ICUs). Incidence was calculated per 1000 patient-days.

The identification of clinical isolates was carried out using conventional methods, including subculturing of positive samples on agar-based solid media and biochemical identification in the Vitek 2 Compact System automated system (bioMerieux, Marcy-l’Étoile, France). Antibiotic sensitivity was tested by using the minimum inhibiting concentration (MIC) method performed by the Vitek 2 Compact system. Gram-negative isolates were tested on the following antibiotics: ampicillin, amoxicillin/clavulanic acid, ampicillin/sulbactam, ticarcillin, piperacillin, piperacillin/tazobactam, cefotaxime, ceftazidime, ceftriaxone, cefepime, aztreonam, ertapenem, meropenem, amikacin, gentamicin, tobramycin, ciprofloxacin, levofloxacin, moxifloxacin, minocycline, tetracycline, tigecycline, colistin, trimethoprim–sulphomethoxazole, ceftolozane/tazobactam, ceftazidime/avibactam, and Fosfomycin. Colistin’s MIC was determined using the reference method, broth microdilution (UMIC^®^ test strips, Bruker, Billerica, MA, USA), as recommended. As far as Gram-positive isolates are concerned, *S. aureus* was tested on cefoxitin, benzylpenicillin, oxacillin, imipenem, gentamicin, ciprofloxacin, moxifloxacin, erythromycin, clindamycin, linezolid, daptomycin, teicoplanin, vancomycin, tigecycline, and trimethoprim–sulphomethoxazole, while *E. faecium* on ampicillin, ciprofloxacin, daptomycin, imipenem, synergy with gentamicin and streptomycin, vancomycin, teicoplanin, and linezolid.

The interpretation of antibiotic resistance was made according to EUCAST guidelines [EUCAST Clinical Breakpoint Tables, version 14.0, valid from 1st January 2024] with isolates categorized as susceptible (including susceptible and increased exposure) or resistant [45].

## 5. Statistical Analysis

Annual trends in antimicrobial resistance (AMR) were analyzed using the Joinpoint Regression Program, version 4.9.1.0 (National Cancer Institute, Bethesda, MD, USA [46]). A segmented log-linear regression model was applied to the log-transformed AMR rates to identify years in which statistically significant changes in trend (“joinpoints”) occurred. For each segment, the annual percentage change (APC) and 95% confidence intervals (CIs) were computed, and significance was assessed using Monte Carlo permutation tests with results interpreted at a two-sided significance level of 0.05. Data were visually inspected for outliers. The APC is tested to determine whether a difference exists from the null hypothesis of no change (0%). The average annual percentage change (AAPC) was estimated as a weighted average of the segment-specific APCs, providing an overall summary of the trend. APC equals to AAPC when it is constant and there are no joint points (no changes in trend).

## Figures and Tables

**Table 1:** Annual distribution of pathogens.

Year	2018	2019	2020	2021	2022	2023	2024
No of Isolates	1373	1568	1520	2485	1984	1772	1600
*Klebsiella pneumoniae*	388 (28.3)	446 (28.4)	461 (30.3)	807 (32.5)	658 (33.2)	662 (37.4)	552 (34.5)
*Acinetobacter baumannii*	380 (27.7)	461 (29.4)	504 (33.2)	807 (32.5)	539 (27.2)	352 (19.9)	351 (21.9)
*MRSA*	160 (11.7)	174 (11.1)	103 (6.8)	246 (9.9)	198 (10.0)	177 (10.0)	198 (12.4)
*VRE*	88 (6.4)	149 (9.5)	161 (10.6)	232 (9.3)	214 (10.8)	164 (9.3)	204 (12.8)
*Pseudomonas aeruginosa*	202 (14.7)	161 (10.3)	174 (11.4)	225 (9.1)	207 (10.4)	293 (16.5)	239 (14.9)
*Stenotrophomonas maltophilia*	89 (6.5)	89 (5.7)	83 (5.5)	132 (5.3)	104 (5.2)	77 (4.3)	26 (1.6)
*Providencia stuartii*	47 (3.4)	48 (3.1)	11 (0.7)	7 (0.3)	44 (2.2)	22 (1.2)	8 (0.5)
*Proteus. mirabilis*	9 (0.7)	15 (1.0)	6 (0.4)	7 (0.3)	8 (0.4)	7 (0.4)	9 (0.6)
*Escherichia coli*	0	6(0.4)	5 (0.3)	9 (0.4)	1 (0.1)	7 (0.4)	7 (0.4)
other Gram-	10 (0.7)	19 (1.2)	12 (0.8)	13 (0.5)	11 (0.6)	11 (0.6)	6 (0.4)

Values in parentheses represent percentages.

**Table 2:** AAPCs of overall AMR by hospital sector.

Sector	AAPC	Lower CI	Upper CI	*p*-Value
Medical	7.3	3.7	11.3	<0.001 *
ICU	0.6	−18.3	17.5	0.08
Surgical	9.8	−1.8	23.3	0.1

* indicates that *p*-value is statistically significant.

**Table 3:** Annual percent change in the incidence of resistant bacteria in the entire hospital.

Species	Segments of Study Period	APC	Lower CI	Upper CI	*p* Value
*Klebsiella pneumoniae*	2018–2021	37.9	22	70.2	<0.001 *
	2021–2024	−12.6	−26	−2.5	0.003 *
*Acinetobacter baumannii*	2018–2021	36.6	22.8	55.1	<0.001 *
	2021–2024	−28.3	−36.9	−20.4	<0.001 *
*Pseudomonas aeruginosa*	2018–2021	9.1	−0.2	21.3	0.06
	2021–2024	9.1	−0.2	21.3	0.06
*MRSA*	2018–2021	21.5	10.1	50.4	<0.001 *
	2021–2024	−9.8	−25.1	−0.9	0.02 *
*VRE*	2018–2021	41.9	−21.7	106.3	<0.001 *
	2021–2024	−11.2	−31.3	1.8	0.09

* indicates that *p*-value is statistically significant.

**Table 4:** The AAPCs of AMR in blood cultures.

Sector	AAPC	Lower CI	Upper CI	*p*-Value
Entire hospital	7.2	−0.9	17.2	0.06
Medical	9.9	3.7	17.6	<0.001 *
ICU	−3.5	−10.7	2.8	0.2
Surgical	17.3 *	4.9	35.0	<0.001 *

* indicates that *p*-value is statistically significant.

## Data Availability

The original contributions presented in this study are included in the article. Further inquiries can be directed to the corresponding authors.
